# Innovative cultural care training: the impact of flipped classroom methods on critical cultural competencies in psychiatric nursing: a quasi-experimental study

**DOI:** 10.1186/s12912-024-02001-z

**Published:** 2024-05-21

**Authors:** Mahdiyeh Sarvarizadeh, Sakineh Miri, Fatemeh Darban, Jamileh Farokhzadian

**Affiliations:** 1 Reproductive Health, Family and Population Research Center, Kerman University of Medical Sciences, Kerman, Iran; 2 Health in Disasters and Emergencies Research Center, Institute for Futures Studies in Health, Kerman University of Medical Sciences, Kerman, Iran; 3 Department of Nursing, School of Medicine, Iranshahr University of Medical Sciences, Iranshahr, Iran; 4 Nursing research center, Kerman University of Medical Science, Kerman, Iran

**Keywords:** Culture, Culturally competent care, Culturally congruent care, Cultural competence, Psychiatric nurses, Mental health care

## Abstract

**Introduction:**

Healthcare systems are encountering a growing number of diverse and multicultural clients due to globalization and migration. To effectively address the challenges associated with cross-cultural interactions, nurses require a comprehensive framework of critical cultural competencies. One potential approach to enhancing these competencies in mental health care settings is to use innovative methods such as the flipped classroom in cultural care training programs. This study evaluated the effect of using the flipped classroom method in cultural care training on the critical cultural competencies of nurses working in a psychiatric hospital.

**Methods:**

This quasi-experimental study involved 70 nurses working in a psychiatric hospital affiliated with the Kerman University of Medical Sciences in southeastern Iran. Through random sampling, the nurses were allocated into two groups of intervention (*n* = 35) and control (*n* = 35). The intervention group participated in a cultural care training program using the flipped classroom method, which consisted of four sessions conducted over a four-week period. To evaluate the nurses’ critical cultural competence, the Almutairi’s Critical Cultural Competence Scale was administered before the training and again one month later.

**Results:**

The study findings indicated no significant differences in the scores of critical cultural competencies between the intervention group (4.53 ± 0.64) and the control group (4.73 ± 0.42) during the pre-test stage (t = 1.53, *p* = 0.13). Both groups had a similar perception of critical cultural competencies, which was not particularly positive. However, in the posttest stage, the intervention group (5.33 ± 0.49) demonstrated a significant increase in critical cultural competencies compared to the control group (4.75 ± 0.44) (t = 5.14, *p* = 0.001).

**Conclusion:**

The study results indicated that the use of the flipped classroom method in the cultural care training program effectively enhanced the critical cultural competencies of nurses. Given the importance of cultural care in both physical and psychiatric care settings for multicultural clients, it is crucial for nurses to receive ongoing in-service education that utilizes innovative and active methods such as the flipped classroom.

## Introduction

The challenges brought about by globalization and immigration have prompted healthcare systems to adapt to diverse and multicultural populations. It is crucial for healthcare providers to provide culturally sensitive services, not only to enhance the quality of care but also to prevent discrimination against culturally diverse populations [1]. Moreover, the medical tourism industry requires significant growth, particularly in developing Asian countries like Iran. Nurses, nursing students, and nurse educators play a crucial role in the advancement and success of this industry. It is essential for these stakeholders to enhance their scientific capabilities and cultural competencies and deliver services that meet the needs of culturally diverse clients, including families, individuals, communities, and populations [2].

Nowadays, there is a growing recognition of providing cultural care to clients with mental illnesses in mental healthcare settings [3]. Nurses working in psychiatric hospitals play a key role in delivering cultural care and affordable mental healthcare to vulnerable populations with mental illnesses [4]. The majority of patients admitted to psychiatric departments suffer from conditions such as schizophrenia, bipolar disorder, and major depressive disorder. During the acute phase of their illness, these patients are hospitalized in the psychiatric ward, where they often exhibit aggressive behaviors, communication and cultural challenges, and suicidal thoughts. The involuntary and forced nature of their hospitalization further complicates effective communication between patients and nurses, making the atmosphere in psychiatric departments distinct from other units [5].

Patients with mental illnesses may struggle to recognize their cultural needs and understand culturally specific nursing interventions that could benefit their mental health [6]. They are often highly sensitive and have a low tolerance for misunderstandings between themselves and the healthcare providers. Many of these patients resort to psychiatric hospitals due to a lack of understanding and stigmatization in social environments. It is crucial for psychiatric nurses to have a deep understanding of their patients’ cultures and provide culturally congruent care [7]. Effective communication skills with patients from diverse cultures are essential for psychiatric nurses to understand their clients’ needs and adapt nursing interventions accordingly to promote their well-being [8].

Campinha-Bacote (2002) developed a model of cultural competence for psychiatric and mental health nursing known as “The Culturally Competent Model of Care”. According to this model, cultural competence is defined as the ongoing process in which the healthcare providers strive to effectively work within the cultural context of their clients, individuals, or communities. It emphasizes that psychiatric nurses should perceive themselves as becoming culturally competent rather than considering themselves culturally competent. This model identifies cultural awareness, cultural knowledge, cultural skill, cultural encounters, and cultural desire as key constructs of cultural competence. All five constructs are essential in delivering interventions that are culturally competent [9].

However, many nurses lack the necessary cultural competence and self-confidence to effectively address the distress and mental health issues faced by patients with mental illnesses. This deficiency in cultural competence among mental health nurses hampers their ability to provide high-quality cultural care to multicultural clients [6]. As a result, healthcare disparities and inequities arise, compromising the physical, psychological, spiritual, social, and cultural well-being of patients, their families, and the healthcare providers [10]. Psychiatric nurses who are unaware of the influence of their own cultural values run the risk of cultural imposition, which involves imposing their beliefs, values, practices, and behavior patterns on another culture [9].

## Theoretical framework

To address challenges mentioned above, Almutairi et al. have introduced the concept of critical cultural competence to assist healthcare providers in managing the complexities and obstacles to cross-cultural interactions with culturally diverse clients [10–12]. The critical cultural competence model, proposed by Almutairi et al. (2015), consists of four components: critical awareness, critical knowledge, critical skills, and critical empowerment. These components include the cognitive, behavioral, and emotional domains. Critical awareness and critical knowledge fall within the cognitive domain, critical skills pertain to the behavioral domain, and critical empowerment resides in the emotional domain [11].

Critical cultural competence expands upon the existing concept of cultural competence found in the literature, with a focus on the critical aspect. This criticality refers to nurses’ ability to make informed decisions based on clinical cases, extending beyond mere knowledge and encompassing skills and attitudes as well. The researchers recommend that organizations with multicultural workforces or culturally diverse clients incorporate the Almutairi’s critical cultural competence model into their new nursing education programs and continuous education initiatives. By integrating this model into training and support programs, healthcare organizations can foster a deeper understanding and appreciation of critical cultural competence among nurses. This, in turn, facilitates the development of nurses’ critical cultural competence [10–12].

Moreover, experts in the field of critical cultural competence argue that incorporating active educational methods that specifically target cognitive, functional, and emotional skills can be more effective in teaching critical cultural competence [13]. These methods aim to create more accessible and practical learning conditions that enhance the acquisition and application of critical cultural competence [11].

The flipped classroom is an active teaching method that has gained popularity in recent years, particularly with the advancement of technology. In a flipped classroom, learners are encouraged to watch video conferences and educational content at home, engaging in individualized and self-directed learning to prepare for collaborative sessions. Subsequently, learners come together in joint sessions to discuss and explore the learned contents interactively. This approach promotes a higher level of learning as individuals have the opportunity to engage in face-to-face discussions and exchange opinions [14]. The flipped classroom model increases teaching flexibility and transforms the roles of both teachers and learners. Learners shift from passive recipients of information to active participants, while instructors transition from leaders and lecturers to supporters and mentors. Additionally, the availability of a wide range of resources allows learners to participate and interact with their instructors [15].

## Review of the literature

The review of the literature indicated that the use of a flipped classroom in teaching was more successful than traditional lecture-based teaching, particularly in adult education. This approach has shown great efficacy in multidisciplinary or clinical training, as well as practical training that focuses on skill development and emotional aspects of learning [14, 16]. In another study, researchers implemented a cultural competence training program for clinical nurses using various methods. These methods included incorporating expert experiences with immigrants and culturally diverse clients, providing books showcasing different cultures, engaging in storytelling, film screenings, role-playing, and drawing real-life scenarios. The training program successfully resulted in a significant improvement in the cultural competence of the nurses. The authors emphasized the importance of using different methods in training programs to develop cultural competence through interactions in diverse situations. These findings serve as a valuable reference for designing the content of in-service cultural competence training for nurses [17]. Other studies have explored the effect of cultural competence training programs on nurses, undergraduate students, postgraduate students, and nurse educators, but they have not used the flipped classroom method and often do not target nurses working in psychiatric settings [2, 18–20]. However, no study specifically investigated the effect of using the flipped classroom method in cultural care training programs on the cultural competencies and critical cultural competencies of nurses, especially those working in psychiatric settings.

### Problem statement and aim

With the process of globalization and the increasing cultural diversity among healthcare users in mental health settings, nurses working in mental health settings are facing new demands and challenges. Assessing and developing critical cultural competence is crucial in effectively addressing the diverse needs of patients with mental illness and fostering trusting relationships between patients and nurses. It is clear from the aforementioned discussion that cultural care training has a profound impact on nurses’ cultural competence. However, most studies in this area have focused on traditional training methods, neglecting active approaches such as the flipped classroom method. Moreover, there is a lack of research on nurses’ critical cultural competence levels and the effectiveness of continuing education with active training programs in increasing their competence. Therefore, interventional studies on cultural care are required to improve nurses’ critical cultural competence in different contexts and cultures. In this study, we aimed to address this gap by designing, implementing, and evaluating a cultural care training program. Specifically, we investigated the effect of using the flipped classroom method in cultural care training on the critical cultural competencies of nurses working in a psychiatric hospital.

### Research hypotheses

Alternative hypothesis (H1): The implementation of flipped classroom method in cultural care training will significantly enhance critical cultural competencies among nurses working in a psychiatric hospital.

Null hypothesis (H0): The implementation of flipped classroom method in cultural care training will not significantly enhance critical cultural competencies among nurses working in a psychiatric hospital.

## Methods

### Study design

In this quasi-experimental study, a pretest-posttest design was used, involving both an intervention group and a control group. The study was conducted in a psychiatric hospital affiliated with Kerman University of Medical Sciences, located in southeastern Iran.

### Target population and sampling

**Fig. 1:**
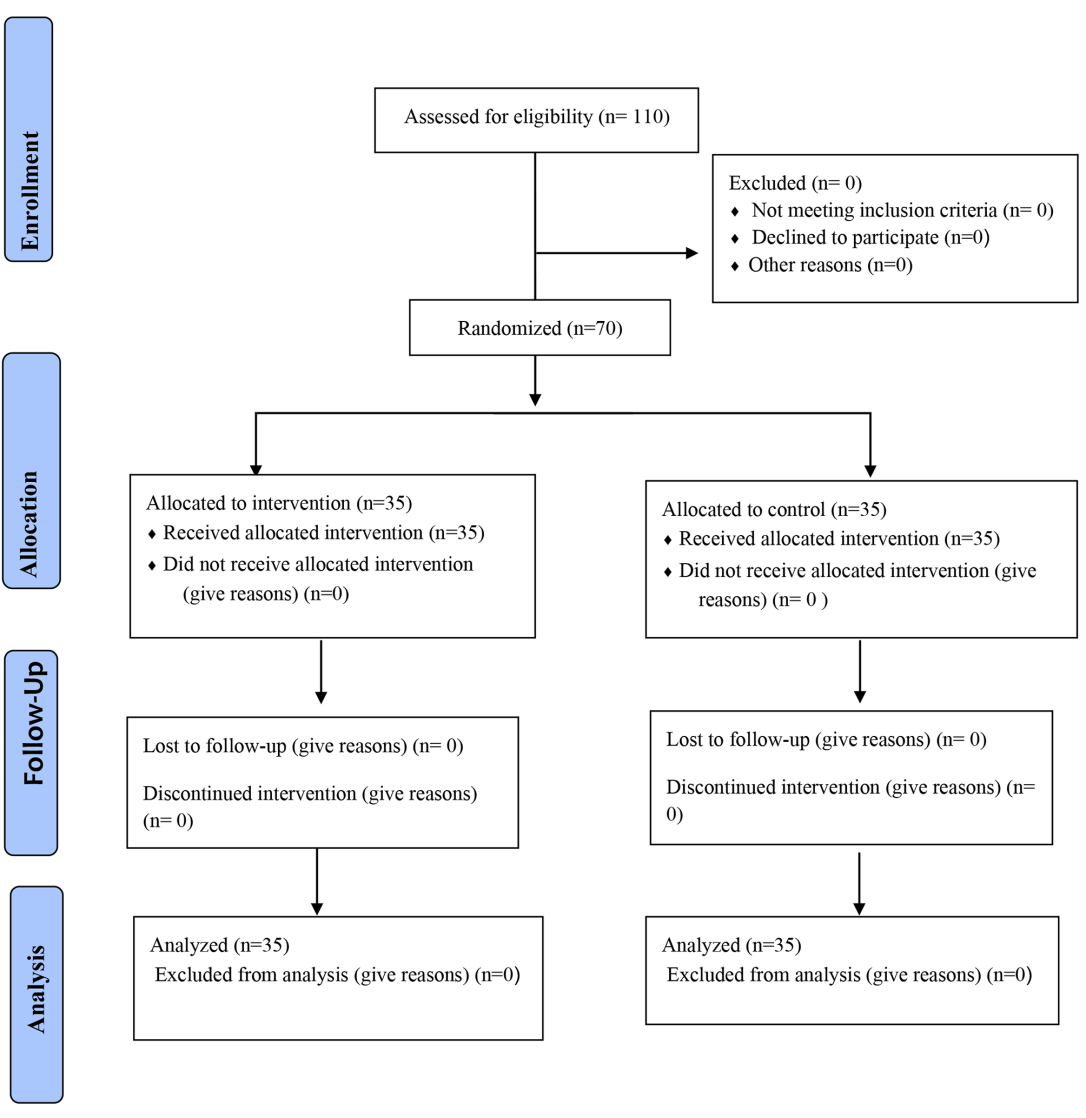
CONSORT flow diagram of participant recruitment, allocation, follow-up, and data analysis

The target population consisted of all nurses working in various departments of the psychiatric hospital (*N* = 110). The sample size was calculated according to a pervious study [2] and the sample size formula. Regarding α = 0.05, a test power of 90%, and a large effect size (Cohen d = 0.7), a sample size of 19 was required for each of the intervention and control groups. However, for increased certainty, 35 were selected for each group, resulting in a total sample size of 70. Random selection and allocation were performed sequentially for the intervention (*n* = 35) and control groups (*n* = 35) using a random number table (Fig. 1).

The inclusion criteria included nurses who held a bachelor’s degree or higher in nursing, had a minimum of 6 months of work experience in a psychiatric hospital, and were willing to participate in the training program. The exclusion criteria were nurses who were absent in more than one training session, on leave, or transferred to another hospital.

### Data collection tool

Two data collection tools were used in this study:

1. **Demographic* and professional* Information Questionnaire**: This questionnaire gathered demographic and professional information, including age, gender, marital status, type of employment, type of shift, position, workplace department, work experience, education level, ethnic and racial background, completion of training courses, and the level of care provided for culturally diverse clients in the hospital.
2. **Almutairi’s Critical Cultural Competence Scale (CCCS)**: The CCCS, developed and validated by Almutairi et al. (2013), consists of 43 items divided into four subscales:

- Critical awareness (12 items): This subscale assesses the ability to recognize cultural differences, power dynamics, potential consequences, and personal attitudes and biases.
- Critical knowledge (7 items): This subscale measures the conceptual perception of culture and knowledge related to communication challenges in cross-cultural interactions.
- Critical skills (7 items): This subscale evaluates the proficiency in using cross-cultural communication skills to discuss cultural meanings and make ethical decisions in clinical settings.
- Critical empowerment (17 items): This subscale focuses on healthcare providers’ perceptions of their own empowerment in the workplace, including experiences of disempowerment due to factors such as race, culture, gender, or economic status [10, 12, 13].

Participants responded to the items using a 7-point Likert scale, ranging from 1 (strongly disagree/never) to 7 (strongly agree/always). Negative item stems were reverse-scored. The total score of the scale was calculated based on the Likert scale, with scores ranging from 1 to 7. A cutoff point of ≥ 5 indicated positive critical cultural competence. The validity of the original version of CCCS was established by the faculty members through the content validity, as well as construct validity through convergent and discriminant validity. The scale demonstrated good reliability, with internal consistency measured using Cronbach’s alpha coefficients of 0.86 for the whole scale, and 0.60, 0.70, 0.77, and 0.77 for critical awareness, critical knowledge, critical skills, and critical empowerment, respectively [10, 12, 13].

In this study, the Persian version of the CCCS was validated through a cultural adaptation, including translation and back-translation, with permission obtained from the original designers. The content validity of the scale was qualitatively assessed by 10 faculty members from the School of Nursing and Midwifery. The reliability of the scale was evaluated using internal consistency, and the Cronbach’s alpha coefficients for cultural awareness, cultural knowledge, cultural skills, cultural empowerment, and the whole scale were 0.74, 0.75, 0.92, 0.88, and 0.83, respectively.

### Intervention procedure and evaluation period

The educational content was prepared based on a comprehensive literature review [2, 12, 13, 17]. This content was carefully reviewed and approved by two faculty members from the nursing school (see Table 1). To facilitate coordination during the implementation of the training program, the first researcher created separate groups for the control and intervention groups in the Rubika application. The first researcher personally provided the consent forms and pre-test questionnaires to both the control and intervention groups two weeks prior to the training. The training program consisted of four two-hour sessions, conducted once a week over a four-week period. The research team members delivered the training program using the flipped classroom method, which involved three stages: activities before the class, activities during the class, and activities after the class.

### Activities before the classroom

One week prior to each educational session, relevant educational materials such as PowerPoint presentations, videos, case reports, stories, and examples of nurses’ experiences, along with the audio file of the content study guide, were made available through the Rubika application. The participants in the intervention group were instructed to independently study the shared content to familiarize themselves with the concepts and fundamentals of each topic. They were also encouraged to write down any problems they encountered. In the flipped classroom approach, the traditional roles of teaching in class and completing homework were reversed. Learners were expected to read the educational materials at home before each class. During the class time, the focus shifted towards solving exercises, working on projects, and engaging in discussions.

### Activities during the classroom

The joint training sessions took place in the hospital training hall. At the beginning of each session, the instructor administered a brief exam consisting of multiple-choice questions related to the subject matter. This was done to ensure that participants had read and understood the content. Following the exam, the instructor delivered a mini lecture accompanied by a PowerPoint presentation, aimed at practicing the topic and addressing any questions or issues raised by the participants. Participants were required to present their group assignments. The instructor encouraged participants to share real-life stories related to the topics and express their personal opinions. Each session lasted approximately 2 h.

### Activities after the classroom

After each session, the instructor encouraged participants to share their opinions and insights using the Rubika application. This allowed the researcher and others to access the content. Additionally, materials were shared with participants to facilitate their preparation for the next session [15].

The nurses were followed up one month after the completion of the training program. The first researcher provided them with necessary explanations regarding data collection through the Rubika application. Post-test questionnaires were personally distributed to both the intervention and control groups, and the nurses completed the questionnaires through self-reporting.

**Table 1:** Themes covered in the cultural care training curriculum

Goal: To improve the nurses’ critical cultural competencies in the psychiatric hospital		
**Target group**: Nurses in the intervention group (*n* = 35) **Expected duration**: Four two-hour sessions in four weeks **Training methods**: The flipped classroom method using lectures and tutorials, PowerPoint presentations, case reports sharing, video clips and short movies, audio-visual materials, group discussion, contributions of work-based experiences, questions and answers **Evaluation method**: Comparison of pre-test and post-test scores of CCCS		
**Sessions**	**Main topic**	**Content**
1	-Objectives, the importance of teaching cultural care -The concepts of cultural care, and cultural diversity	-The necessity of multicultural education in higher education, factors affecting cultural diversity -Definition of culture, the iceberg of culture, and the components of culture such as age, religion, accent and language, and roles related to gender identity. - Definition of ethnicity, nationality, and race - Migration, culturally diverse immigrants - Needs of culturally diverse clients - An overview of the culture of Kerman, southeastern Iran, and cultural diversity (accent, clothing and eating patterns, religion) - Provision of examples and cases, and introduction of a book
2	-Cultural competence and critical cultural competence -Integration of cultural care into the nursing process	- Definition of cultural competence and critical cultural competence - The importance of developing cultural competence and critical cultural competence in mental healthcare providers -Definition of organizational cultural competence and how to develop cultural competence in the organization and its obstacles - Stages of the nursing process -Assessing clients’ cultures using the LEARN and 4 C models and documenting the health beliefs of clients/their disease and health model, and cultural interview report -Nursing diagnoses and interventions and culturally congruent care standards Evaluation of interventions, and data collection and documentation of cultural care process -Assignments for next sessions: writing examples and cases according to the experiences of nurses, cultural assessment and appropriate interventions for patients according to the nursing process, and study of the articles suggested by the professor
3	-Cultural care and cultural competence models -Critical cultural competence model	- The Leininger’s cultural care theory (cultural protection, cultural adaptation, cultural re-patterning, cultural mediation, competence building) - The Giger and Davidhizar’s transcultural assessment model (communication, space, social organization, time, environmental control, and biological changes) - The Purnell’s model for cultural competence (culture or heritage, communication, family roles, and organization, workforce issues, high-risk behaviors, nutrition, pregnancy, death rituals, spirituality, health care operations, and health care professionals) - The Campinha-Bacote’s model of cultural competence in healthcare delivery (cultural awareness, cultural knowledge, cultural skill, cultural exposure, and cultural desire) -Almutairi’s critical cultural competence model -Assignments for next sessions: presenting a case study dealing with cultural care measures and interventions based on the Leininger’s sunrise model, presenting a patient assessment scenario based on Purnell’s model, and writing your views on the presented video.
4	-Outcomes of cultural competence - Barriers to cultural competence -Provision of cultural care to immigrants	-Positive health outcomes related to cultural competence and provision of cultural care -Barriers to cultural care (stereotyping, prejudice, racism, ethnicism, cultural imposition, cultural conflict, cultural shock), Health inequalities and socioeconomic status, insufficient knowledge and skills of caregivers in establishing transcultural communication, social factors determining health, health equity, social justice, health literacy -Comprehensive scenarios of providing care to immigrants, showing a clip about racism -Playing a role and drawing a real situation for nurses and talking about the impact of these prejudices and judgments on the lives of immigrants -Summary and debriefing.

### Statistical analysis

The data were analyzed using SPSS21. Descriptive statistics such as frequency, percentage, mean, and standard deviation were utilized to summarize the data. The Kolmogorov-Smirnov test was used to assess the normality of the data. Chi square test, or Fisher’s exact test was employed to detect any statistical difference in the demographic variables between the two study groups at baseline. An independent samples t-test was conducted to compare the CCCS scores between the two groups before and after the intervention. Additionally, a paired t-test was used to compare the CCCS scores within each group before and after the intervention. An analysis of covariance was applied to control for the impact of pretest scores on CCCS scores. Multivariate linear regression was used to determine whether demographic variables of the nurses could predict changes in the CCCS score. Effect size estimates were calculated using Cohen’s d to assess the practical or theoretical importance of an effect, as well as the power of the analysis. The significance level was considered ≤ 0.05.

### Ethics approval and consent to participate

The present study (No. 401,000,461) was approved by the Ethics Committee of Kerman University of Medical Sciences (IR.KMU.REC.1401.374). All steps and procedures were performed in accordance with the Declaration of Helsinki and the Committee on Publication Ethics (COPE). Necessary permissions were presented to the hospital prior to the study. At the beginning of the study, all nurses provided written informed consent to participate. The participants were assured of the confidentiality of their information and voluntary participation; they could withdraw from the study at any stage without any negative consequences.

## Results

### Demographic and professional information of nurses

Table 2 shows that 70 nurses (100% response rate) participated in the study. The majority of participants in both the intervention and control groups were female nurses (77.1%, 88.6%), aged 31–40 years (37.1%, 40%), working in men’s wards (42.9%, 68.6%), with permanent employment (68.6%, 74.3%), rotating shifts (94.3%, 88.6%), and a bachelor’s degree (85.7%, 80%). They had 11–20 years of work experience (62.9%, 61.8%), had not attended previous cultural care training (85.7%, 94.3%), and provided moderate levels of cultural care to multicultural clients (51.4%, 48.6%). Both groups shared similar demographic and professional information.

**Table 2:** Comparison of demographic and professional information between the two study groups

Variables	Categories	Intervention		Control		X^2a^	*P*-value ^b^
		n	%	n	%		
Age groups	20–30	9	25.8	8	22.9	0.9	0.95
	31–40	13	37.1	14	40		
	≥ 41	13	37.1	13	37.1		
Gender	Male	3	8.6	6	17.1	1.14	0.28
	Female	32	91.4	29	82.9		
Work position	Nurse	32	91.4	31	88.6	5.6	0.13
	Head nurse	1	2.9	2	5.7		
	Supervisors	2	5.7	2	5.7		
Type of employment	Committed	3	8.6	4	11.4	2.02	0.73
	Temporary-to-permanent	3	8.6	3	8.6		
	Contract recruiters	5	14.9	2	5.8		
	Hired (permanent)	24	68.6	26	74.2		
Shift work	Fixed	2	5.7	4	11.4	0.72	0.39
	Rotating	33	94.3	31	88.6		
Type of ward	Emergency children	0 5	0 14.3	2 3	5.7 8.6	9.34	0.053
	Women	13	37.1	4	11.4		
	Men	15	42.9	24	68.36		
	Supervisory	2	5.7	2	5.7		
Education level	Bachelor	30	85.7	28	80	5.46	0.14
	Master	5	14.3	7	20		
Work experience (years)	˂10	10	28.6	7	20.6	1.53	0.46
	10–20	22	62.9	21	61.8		
	≥ 21	3	8.5	6	17.6		
Attendance at previous training on cultural care	Yes	5	14.3	2	5.7	1.42	0.23
	No	30	85.7	33	94.3		
The level of care for culturally diverse people in clinical settings	Moderately	18	51.4	17	48.6	1.51	0.46
	High	10	28.6	14	40		
	Very high	7	20	4	11.4		
Ethnic and racial background	Native of Kerman province	32	91.4	29	82.9	3.14	0.2
	Others	3	8.6	6	17.1		

Note. ^a^: chi-square/fisher exact test, and ^b^: Bold *p*-values are significant at level of ≤ 0.05

### Outcomes

#### Comparison of changes in the critical cultural competencies

Table 3 presents the levels of critical cultural competencies and scores for four CCCS subscales in both the intervention and control groups before and after the training program. Prior to the program, there was no significant difference in the scores of critical cultural competencies between the intervention group (4.53 ± 0.64) and the control group (4.73 ± 0.42). Both groups had no positive perception of critical cultural competencies based on the CCCS scoring (t = -1.53, *p* = 0.13).

However, after the training program, the intervention group (5.33 ± 0.49) showed a significant increase in the score of critical cultural competencies compared to the control group (4.75 ± 0.44), with a large effect size (Cohen d = 1.22, t = 5.14, *p* = 0.001). According to the CCCS scoring, the intervention group exhibited a positive perception of critical cultural competencies.

Furthermore, the intervention group’s total scores of CCCS and its subscales improved after the training program, with effect sizes ranging from 0.8 to 1.5. Based on the CCCS scoring, the intervention group demonstrated a positive perception of critical cultural competencies.

**Table 3:** Comparison of critical cultural competencies scores between control and intervention groups before and after the training program

	Time	Pre-intervention	Post-intervention				
Variables	Groups	M ± SD	M ± SD ^a^	Within group differences	ES (Cohen d)	Paired *t*-test	*P*- value ^**b**^
Cultural awareness	Intervention	4.29 ± 0.97	5 ± 0.75	0.7	0.8	-4.11	**0.001**
	Control	4.46 ± 0.91	4.62 ± 0.6	0.15	0.2	-1.07	0.29
	Independent *t*- test	0.74	2.27				
	*P*-value	0.45	**0.02**				
	ES^*^ (Cohen d)	0.17	0.54				
Cultural knowledge	Intervention	4.38 ± 0.88	5.48 ± 0.53	1.09	1.5	-6.71	**0.001**
	Control	4.54 ± 0.87	4.65 ± 0.96	0.11	0.12	-0.60	0.55
	Independent *t*- test	-0.73	4.42				
	*P*-value	0.46	**0.001**				
	ES^*^ (Cohen d)	0.17	1.05				
Cultural skills	Intervention	4.29 ± 1.36	5.58 ± 1.27	1.28	0.97	-4.30	**0.001**
	Control	4.7 ± 1.07	4.71 ± 1.2	0.008	0.007	-0.06	0.95
	Independent *t*- test	-1.40	2.92				
	P-value	0.16	0.005				
	ES^*^ (Cohen d)	0.33	0.69				
Cultural empowerment	Intervention	4.6 ± 1.11	5.39 ± 0.68	0.78	0.85	-3.66	**0.001**
	Control	5 ± 0.62	4.89 ± 0.59	0.11	0.18	1.31	0.19
	Independent *t*- test	-1.85	3.25				
	*P*-value	0.06	**0.002**				
Total of critical cultural competence	Intervention	4.53 ± 0.64	5.33 ± 0.49	0.79	1.4	-6.34	**0.001**
	Control	4.73 ± 0.42	4.75 ± 0.44	0.02	0.47	-0.33	0.73
	Independent *t*- test	-1.53	5.14				
	P-value	0.13	0.001				
	ES^*^ (Cohen d)	0.36	1.22				

Note. ^a^: SD, Standard deviation, and M, Mean. The cutoff point of mean ≥ 5 indicates positive critical cultural competence

^b^: Bold p-values are significant at a level of ≤ 0.05

ES: Effect size, and ES of 0-0.2 = small effect, ES of 0.2–0.5 = moderate effect, ES of > 0.5–0.7 = large effect, and ES of > 0.7 = very large effect

An analysis of covariance was performed to control for the effects of the pre-test on the post-test scores of critical cultural competencies. The results indicated that the significant increase in the critical cultural competencies of the intervention group was attributed to the cultural care training (Table 4). These findings corroborate the results presented in Table 3.

**Table 4:** Result of covariance analysis for the two groups of control and intervention

		Type III sum of squares	df	Mean square	F	*p*-value ^a^
Cultural awareness	Intercept	40.5	1	40.5	97.01	**0.001**
	Pretest	4.26	1	4.26	10.22	**0.002**
	Group	3.04	1	3.04	7.28	**0.009**
	Error	27.97	67	0.41		
Cultural knowledge	Intercept	45.47	1	45.47	77.01	**0.001**
	Pretest	1.82	1	1.82	3.12	0.082
	Group	12.64	1	12.64	21.42	**0.001**
	Error	39.55	67	0.59		
Cultural skills	Intercept	54.1	1	54.1	40.53	**0.001**
	Pretest	15.73	1	15.73	11.79	**0.001**
	Group	18.07	1	18.07	13.54	**0.001**
	Error	89.42	67	1.33		
Cultural empowerment	Intercept	42.58	1	42.58	108.11	0.**001**
	Pretest	1.8	1	1.8	4.57	**0.036**
	Group	5.46	1	5.46	13.88	**0.001**
	Error	26.38	67	0.39		
Total of critical cultural competence	Intercept	12.46	1	12.46	63.05	**0.001**
	Pretest	1.79	1	1.79	9.09	**0.004**
	Group	6.87	1	6.87	34.78	**0.001**
	Error	13.24	67	0.19		

Note. a: Bold p-values are significant at level of ≤ 0.05

### Multivariate linear regression

After conducting multivariate linear regression, it was found that the demographic variables of nurses could not significantly predict changes in the critical cultural competencies scores. Consequently, the effectiveness of the training program was not dependent on the nurses’ demographic variables.

## Discussion

### Principal results

This study aimed to evaluate the effect of using the flipped classroom approach in a cultural care training program on the critical cultural competencies of nurses working in a psychiatric hospital. The results indicated a significant improvement in nurses’ perception of critical cultural competencies across all dimensions. Remarkably, no interventional studies have investigated the impact of educational approaches such as the flipped classroom in cultural care training on critical cultural competence in mental health care settings. Consequently, this study relied on descriptive studies investigating the level of critical cultural competencies [12, 18] and interventional and systematic review studies investigating the effects of various training methods on cultural competencies in healthcare providers in various settings [2, 17, 19–25].

Almutairi et al. (2017) conducted a study in Canadian hospitals and found positive perceptions of critical cultural competence among nurses. They also identified age, work experience, and birthplace as factors influencing nurses’ perception of critical cultural competence. Developing cultural competence requires exposure to diverse patient populations and the acquisition of cultural knowledge and awareness. Healthcare organizations should implement ongoing cultural training programs to enhance the cultural competence of nursing staff and address challenges in cross-cultural interactions [12]. Wang et al. (2022) found that Chinese nurses exhibited a less desirable level of critical cultural competence. However, female nurses, those working in intensive care, and those in higher positions demonstrated higher levels of critical cultural competence. The authors suggested targeted training using active methods to strengthen the critical cultural competence of nurses. It is crucial not to overlook male and lower-ranking nurses, as they also require encouragement to participate in cultural competence training programs [18].

Park et al. (2019) examined the effects of a cultural nursing course on enhancing the cultural competence of nursing students in Korea. The course employed various methods, including small group activities, discussions, presentations, experiential learning, reflective activities, and lectures. Upon completion of the course, the students showed a noticeable increase in their cultural competence [19]. A systematic review conducted by Oikarainen et al. (2019) examined the impact of educational interventions on the development of nurses’ cultural competence. The review encompassed studies that utilized various approaches, including traditional training, web-based modules, lectures, group discussions, case studies, reflective exercises, and simulation. The findings indicated that these interventions effectively increased the cultural competence of nurses in the intervention group. The researchers emphasized the importance of prioritizing the development of cultural competence among nurses and nursing students through education, as nurses frequently interact with culturally diverse clients in various healthcare settings. They also highlighted the need for more high-quality studies to investigate the effectiveness of educational interventions in enhancing nurses’ cultural competence. Furthermore, future research should focus on reporting specific components of interventions and employing effective, proactive, and innovative methods [20].

Studies conducted in Iran have also supported the effectiveness of virtual cultural care training in enhancing the cultural competence of undergraduate students [22], postgraduate students [2], and nurse educators [21]. A review study (2019) focused on fostering cultural competence in nursing education through a values-based learning approach. The review emphasized the significance of incorporating the six core values of nursing (caring, compassion, commitment, communication, courage, and competence) into the undergraduate nursing curriculum to prepare nurses for culturally competent care. The authors suggested that exploring the underlying values of culturally sensitive care required a revision of educational philosophies and the implementation of innovative learning-teaching methods, both in the classroom and in clinical environments [23].

Stone et al. (2018) evaluated the effectiveness of a one-day workshop aimed at enhancing cultural competence among Chinese nursing students. The workshop was designed based on the Mezirow’s transformative learning theory and incorporated various educational strategies. These strategies included intensive educational lectures and a series of self-reflection activities such as watching videos, drawing a cultural self-portrait, and participating in a social attitude implicit association test. The results showed a significant increase in participants’ scores in the components of cultural awareness, cultural knowledge, cultural perception, and cultural skills within one month and three months after the intervention. However, there was no significant difference in cultural respect before and after the intervention [24].

In contrast, Kula et al. (2021) conducted a study in Israel to investigate the impact of cultural competence training on nursing students in emergency situations. The findings revealed that while the training increased cultural knowledge, it did not significantly enhance nurses’ cultural skills and attitudes in emergency situations. Consequently, the online training did not instill enough confidence in participants to apply the acquired techniques in real emergency environments. The researchers recommended further studies in this field using different methods and encompassing culturally diverse contexts [25].

The differences between our study and the previously mentioned study can be attributed to several factors, including variations in the research environment, the tools employed, the training methods utilized, the unique working conditions in emergency departments, the cultural diversity among emergency patients, the acute condition of patients due to the increased workload of nurses, and limited time available for practicing the educational content. Most of the aforementioned studies focused on educational interventions for nursing students and nurses, with the exception of those in mental healthcare settings. However, there is a clear need for more studies in this field, particularly among nurses in mental healthcare.

### Limitations and strengths

The present study had certain limitations which should be taken into consideration when interpreting the results. Firstly, since this study was conducted in a psychiatric hospital, contamination between two groups was unavoidable and may have affected the results. To reduce contamination, nurses in the intervention group were informed about its disadvantages and were instructed not to share the training or evaluation with other colleagues. However, differences in results between the two groups indicated that the intervention was still effective even with contamination. Secondly, the results were specific to nurses working in a psychiatric hospital in southeastern Iran, and the evaluation of the training program’s effectiveness relied on self-reported measures. Moreover, due to the heavy shift work of nurses and time and financial constraints, data collection for the evaluation step was conducted over a one-month interval. Therefore, caution should be exercised when generalizing the findings. Another limitation of the study was the lack of data on feasibility of implementing the training program and nurses’ satisfaction with the training program. It is recommended to extend the duration of the training programs to cover more topics and employ multiple methods for assessing competencies and conducting follow-up evaluations. Additionally, future studies should provide a more comprehensive understanding of the long-term impact of the training on competencies, as well as explore feasibility and acceptability of training programs, and nurses’ satisfaction with such programs in different sociocultural conditions. It would be beneficial for future research to focus on disseminating such novel training more widely and overcoming organizational barriers to facilitate higher levels of critical cultural competencies among healthcare providers.

Despite these limitations, the study had notable strengths. The experiences gained from designing and implementing the cultural care training program using the flipped classroom method can be valuable for nurse educators. Furthermore, the validation and translation of the Persian version of the CCCS in this study provide a valuable tool for evaluating critical cultural competencies among nurses and other healthcare providers in the Iranian context.

## Conclusions

The study results indicated that the use of the flipped classroom method in the cultural care training program successfully improved the critical cultural competencies of nurses. Considering the significance of cultural care in meeting the needs of diverse clients in various healthcare settings, it is essential for nurses to receive continuous education and training. Innovative and active approaches such as the flipped classroom can be effective in providing this necessary training. Nurse educators have the opportunity to develop cultural care training programs using innovative and active approaches such as the flipped classroom for fostering critical cultural competencies among nurses and nursing students.

### Implications

The study results have important practical implications for mental healthcare providers, educators, researchers, and administrators. Future researchers should consider using qualitative and innovative methods such as Online Photovoice (OPV) [26–28], Online Interpretative Phenomenological Analysis (OIPA) [29], and Community-Based Participatory Research (CBPR) [30] to further investigate the same topic. These approaches will help capture the thoughts, feelings, images, and behaviors of individuals from their own unique experiences, leading to more grounded research and the development of more effective services. Using OPV, OIPA, and/or CBPR allows to explore the impact of flipped classroom training on critical cultural competencies of nurses in both physical care and psychiatric hospitals. Involving community members such as nurses, students, and people in the research process will provide valuable insights into various related topics. OPV, in particular, allows participants to express their experiences with minimal manipulation compared to traditional quantitative methods. Furthermore, future researchers can explore the use of OPV in qualitative or mixed-method studies to explore the impact of flipped classroom training on critical cultural competencies of nurses in psychiatric hospitals. Educators and trainers can also utilize OPV for experiential activities to enhance group and organizational synergy.

## Data Availability

The data are available upon request to the corresponding author after signing appropriate documents in line with ethical applications and the decision of the ethics committee.

## Abbreviations

CCCS: Critical Cultural Competence Scale
OPV: Online Photovoice
OIPA: Online Interpretative Phenomenological Analysis
CBPR: Community-Based Participatory Research
